# Two-component regulatory systems in *Helicobacter pylori* and *Campylobacter jejuni*: Attractive targets for novel antibacterial drugs

**DOI:** 10.3389/fcimb.2022.977944

**Published:** 2022-08-24

**Authors:** Javier Casado, Ángel Lanas, Andrés González

**Affiliations:** ^1^ Group of Translational Research in Digestive Diseases, Institute for Health Research Aragón (IIS Aragón), Zaragoza, Spain; ^2^ Department of Biochemistry and Molecular & Cellular Biology, University of Zaragoza, Zaragoza, Spain; ^3^ Department of Medicine, Psychiatry and Dermatology, University of Zaragoza, Zaragoza, Spain; ^4^ Biomedical Research Networking Center in Hepatic and Digestive Diseases (CIBERehd), Madrid, Spain; ^5^ Digestive Diseases Service, University Clinic Hospital Lozano Blesa, Zaragoza, Spain

**Keywords:** two-component regulatory system, *Epsilonproteobacteria*, *Helicobacter pylori*, *Campylobacter jejuni*, orphan response regulator, therapeutic target, *Campylobacterota*

## Abstract

Two-component regulatory systems (TCRS) are ubiquitous signal transduction mechanisms evolved by bacteria for sensing and adapting to the constant changes that occur in their environment. Typically consisting of two types of proteins, a membrane sensor kinase and an effector cytosolic response regulator, the TCRS modulate *via* transcriptional regulation a plethora of key physiological processes, thereby becoming essential for bacterial viability and/or pathogenicity and making them attractive targets for novel antibacterial drugs. Some members of the phylum *Campylobacterota* (formerly *Epsilonproteobacteria*), including *Helicobacter pylori* and *Campylobacter jejuni*, have been classified by WHO as “high priority pathogens” for research and development of new antimicrobials due to the rapid emergence and dissemination of resistance mechanisms against first-line antibiotics and the alarming increase of multidrug-resistant strains worldwide. Notably, these clinically relevant pathogens express a variety of TCRS and orphan response regulators, sometimes unique among its phylum, that control transcription, translation, energy metabolism and redox homeostasis, as well as the expression of relevant enzymes and virulence factors. In the present mini-review, we describe the signalling mechanisms and functional diversity of TCRS in *H. pylori* and *C. jejuni*, and provide an overview of the most recent findings in the use of these microbial molecules as potential novel therapeutic targets for the development of new antibiotics.

## Introduction

Phylum *Campylobacterota*, previously known as *Epsilonproteobacteria* (Oren and Garrity, 2021), is a metabolically and physiologically diverse group of prokaryotes comprising both pathogenic and commensal heterotrophic genera such as *Helicobacter*, *Campylobacter*, *Wolinella*, and *Arcobacter*, but also free-living inhabitants of sulphidic ecosystems that are considered important primary producers (Waite et al., 2017). Among pathogenic members of *Campylobacterota*, some species constitute the etiological agents of relevant infections and serious associated-diseases in humans.

*Helicobacter pylori* colonizes human stomach of more than half of the global population (Hooi et al., 2017) inducing chronic inflammation of the gastric epithelium that in some cases progresses to atrophic gastritis, peptic ulcer disease, gastric adenocarcinoma, and mucosa associated lymphoid tissue (MALT) lymphoma (Kao et al., 2016; Chmiela and Kupcinskas, 2019). This microorganism has been associated with 90% of non-cardia gastric cancer worldwide (Moss, 2017) and constitutes the only bacterial pathogen classified as a class I carcinogen (IARC et al., 1994). *Campylobacter jejuni* is recognized as the major cause of bacterial food-borne gastroenteritis worldwide, being responsible of 80-90% of all cases of diagnosed campylobacteriosis (Elmi et al., 2020). The bacterium is part of the commensal microbiota of different avian species and other wild and domestic animals (Burnham and Hendrixson, 2018; Thepault et al., 2020), and it is transmitted to humans mainly by consumption of contaminated meat and other foods (Facciola et al., 2017). Despite the fact that infection in humans usually produces only mild and self-limited bloody diarrhea, severe complications and even death could occur in very young children, elderly people and immunocompromised patients (Same and Tamma, 2018; Igwaran and Okoh, 2019).

Antibiotic chemotherapy is crucial for treatment of *H. pylori* infection and mitigation of gastric cancer incidence (Rokkas et al., 2017; Leung et al., 2018; Nam et al., 2019); but also to treat invasive or extra-gastrointestinal infections by *C. jejuni* and reducing the risk of severe complications of campylobacteriosis in more susceptible populations (Arguello et al., 2015; Mizuno et al., 2022). However, the increasing development of antibiotic resistance and the rapid propagation of multidrug-resistant strains of these clinically relevant pathogens worldwide have contributed to a concerning detriment of the efficacy of current antibiotic therapies (Boyanova et al., 2019; Mourkas et al., 2019; Kuo et al., 2021; Sciortino et al., 2021). As a consequence, several studies have been focused on the identification and validation of new potential therapeutic targets in these microorganisms that lead to the development of novel and effective antibacterial strategies (Hossain et al., 2018; Deblais et al., 2019; González et al., 2019b; González et al., 2020; Roszczenko-Jasinska et al., 2020; Salillas and Sancho, 2020).

## TCRS: A bacterial signal transduction mechanism to sense, respond, and adapt to environmental changes

**Figure 1:**
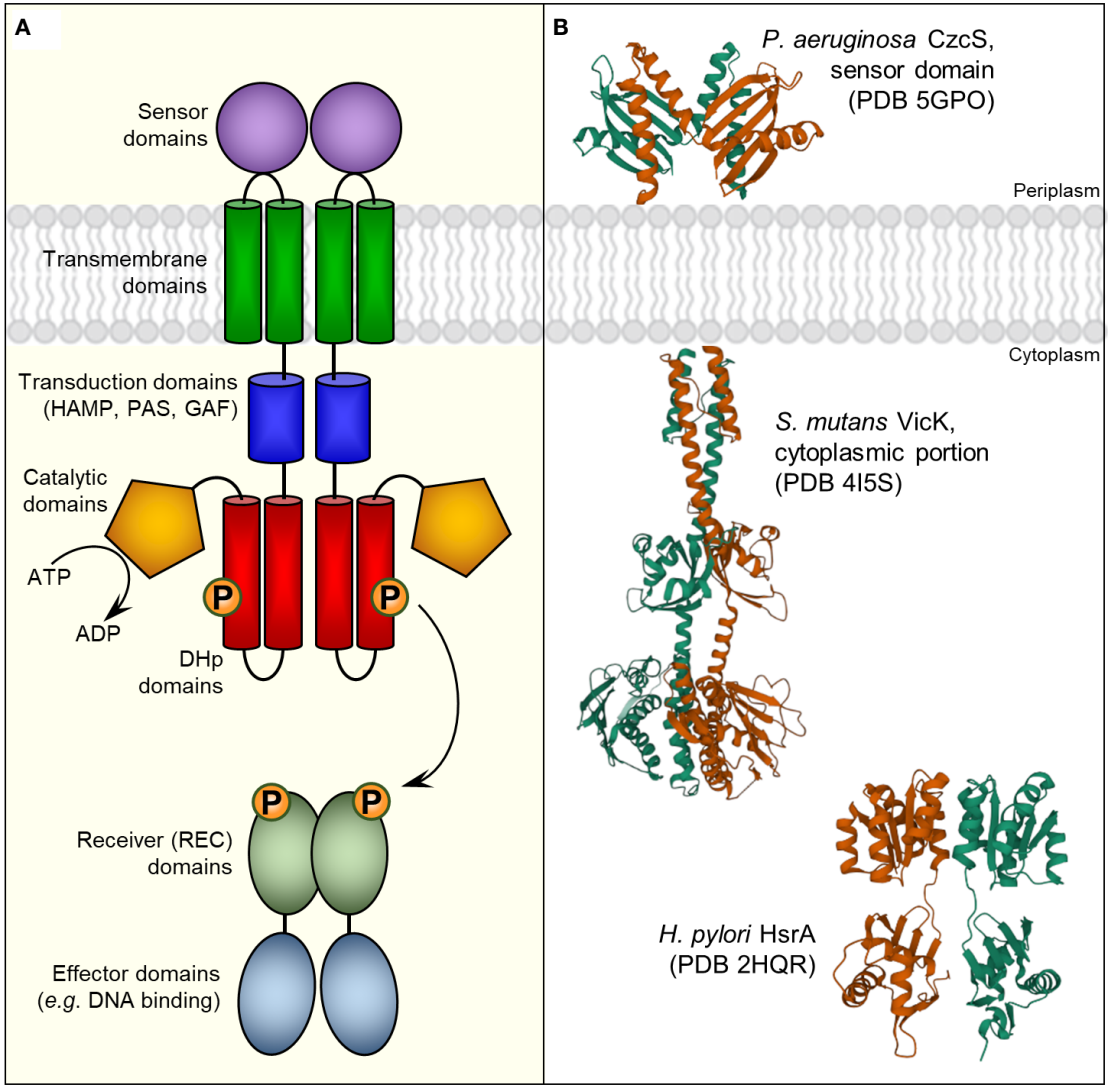
Bacterial two-component regulatory systems (TCRS). **(A)** Schematic representation of a typical TCRS comprising a membrane-anchored sensor histidine kinase **(HK)** and its cognate cytoplasmic response regulator (RR). Although microbial HKs can exhibit a considerable variety in their architectures (Jacob-Dubuisson et al., 2018), a canonical HK consists in a multi-domain homodimeric protein with extracellular sensors that perceives specific stimuli and undergoes autophosphorylation in a conserved His residue through a cytosolic catalytic-ATP binding domain. Phosphoryl group is subsequently transferred to a conserved Asp residue of the cognate RR promoted mainly by intermolecular interactions between the dimerization and histidine phosphotransfer (DHp) domains of the HK and the receiver (REC) domain of the RR (Yamada et al., 2009). Thus, the specificity of the interaction between a given HK and its cognate RR appears to be primarily defined by the nature and positions of contacting residues at the interface between the DHp **(HK)** and the REC (RR) domains, though additional HK domains could also be involved in RR binding (Buschiazzo and Trajtenberg, 2019). **(B)** 3D structures of different HK- and RR-functional domains. Corresponding Protein Data Bank (PDB) entries appear in parentheses.

Two component regulatory systems (TCRS) are ubiquitous signal transduction mechanisms among bacteria that enable microbial cells to perceive both environmental changes and internal information and subsequently trigger a rapid physiological response in order to better adapt to the new conditions. Typically, a TCRS comprises two different proteins that act in concert, a membrane-anchored histidine kinase (HK) and a cognate soluble response regulator (RR), which mostly acts as a DNA binding transcription factor (**Figure 1**). Chemical or physical stimuli are perceived by the HK, which subsequently undergoes autophosphorylation and transduces the signal *via* phosphoryl group to the REC domain of the RR. Phosphorylation usually induces conformational changes in the RR structure that activate or promote their effector functions (Gao et al., 2019). Notably, some RRs appear to act with independence of a cognate HK, which are known as “orphan” RRs. In some cases, these atypical unpaired RRs could naturally lack the conserved Asp and/or other residues required for the phosphotransfer reaction or retain their effector function upon substitution of these residues in the receiver domain, suggesting the existence of phosphorylation-independent mechanisms of regulation (Beier, 2012).

## Structural and functional diversity of TCRS in *H. pylori* and *C. jejuni*

Although the physiological roles of most HKs and RRs predicted in whole-genome sequencing analyses of major pathogenic species of *Campylobacterota* remain poorly understood, significant advances in the comprehension of signalling transduction mechanisms and regulatory functions of certain TCRS have been achieved. As we detail below, strains of *H. pylori* and *C. jejuni* express a variety of TCRS and atypical RRs that control the on/off switch of a plethora of genes and operons involved in relevant physiological processes and virulence mechanisms (**Table 1**). Notably, the amount and complexity of TCRS in these host-associated *Campylobacterota* species are far from those observed in predicted signaling systems of free-living microorganisms included in the same phylum such as *Sulfurospirillum* spp. or *Sulfurimonas* spp., which exhibit a broader ecological breath. Thus, the extensive adaptation of *H. pylori* and *C. jejuni* to the restricted ecological niches of the gastro-intestinal tracts of their host led to streamlined genomes with a very small number of TCRS and minimal complexity in the signal transduction mechanisms (Mo et al., 2022).

**Table 1:** TCRS expressed by *H. pylori* and *C. jejuni*.

TCRS (ORF)	Confirmed or predicted function	References
***H. pylori***		
ArsR/S (*hp0166-hp0165*)	Acid acclimation, adhesin expression, quorum sensing, biofilm formation	(Pflock et al., 2005; Pflock et al., 2006; Wen et al., 2007; Goodwin et al., 2008; Servetas et al., 2016; Acio-Pizzarello et al., 2017; Servetas et al., 2018)
CrdR/S (*hp1365-hp1364*)	Copper homeostasis, nitrosative stress	(Waidner et al., 2005; Hung et al., 2015)
FlgR/S (*hp0703-hp0244*)	Flagellum biosynthesis, motility	(Spohn and Scarlato, 1999; Brahmachary et al., 2004; Lertsethtakarn et al., 2011)
HsrA (*hp1043*)	Translation, transcription, energy metabolism, nitrogen metabolism, chemotaxis, redox homeostasis	(Olekhnovich et al., 2013; Olekhnovich et al., 2014; Pelliciari et al., 2017)
HP1021 (*hp1021*)	DNA replication, synthesis of Fe-S clusters, acetone metabolism, response to oxidative stress	(Pflock et al., 2007; Donczew et al., 2015; Szczepanowski et al., 2021)
CheY1 (*hp1067*)	Chemotaxis	(Foynes et al., 2000; Jimenez-Pearson et al., 2005; Lertsethtakarn et al., 2011)
CheY2/A (*hp0392*)	Chemotaxis	(Foynes et al., 2000; Jimenez-Pearson et al., 2005)
***C. jejuni***		
BumR/S (*cjj1483-cjj1484*)	Butyrate sensing, basic metabolism, iron/heme acquisition, respiration, colonization factors	(Luethy et al., 2015; Goodman et al., 2020)
RacR/S (*cj1261-cj1262*)	Heat shock response, cell morphology, motility, glutamine catabolism, respiratory metabolism	(Bras et al., 1999; Apel et al., 2012; van der Stel et al., 2015a; van der Stel et al., 2015b)
CprR/S (*cj1227c-cj1226c*)	Biofilm formation, osmotic stress resistance, cell envelope	(Svensson et al., 2009; Svensson et al., 2015)
DccR/S (*cj1223c-cj1222c*)	Chicken colonization	(MacKichan et al., 2004; Wosten et al., 2010)
PhosR/S (*cj0890c-cj0889c*)	Phosphate uptake mechanisms, Pho regulon	(Wosten et al., 2006)
FlgR/S (*cj1024c-cj0793*)	Flagellum biosynthesis, motility	(Wosten et al., 2004; Hendrixson, 2006; Boll and Hendrixson, 2013)
CosR (*cj0355c*)	Oxidative stress defenses, respiration, energy metabolism and biosynthesis, gene regulation, copper tolerance, flagellum biosynthesis, lipid metabolism, biofilm formation, efflux pumps	(Hwang et al., 2011; Hwang et al., 2012; Grinnage-Pulley et al., 2016; Park et al., 2021)
CbrR (*cj0643*)	Sodium deoxycholate resistance, flagellum biosynthesis, biofilm formation, chemotaxis	(Raphael et al., 2005; Parrish et al., 2007; Cox et al., 2022)
CheY (*cj1118c*)	Chemotaxis	(Lertsethtakarn et al., 2011; Zautner et al., 2012)
CheA (*cj0284c*)	Chemotaxis	(Lertsethtakarn et al., 2011; Zautner et al., 2012)

### Helicobacter pylori

One of the best-studied TCRS in this gastric pathogen contributes to acid acclimation and is required for gastric colonization. The ArsRS signal transduction mechanism is composed of the sensor ArsS HK and its cognate OmpR-like ArsR RR, which are co-transcribed and negatively auto-regulated by phosphorylated ArsR (Dietz et al., 2002). ArsS-dependent phosphorylation increases the affinity of ArsR by promoters of a variety of genes involved in acid acclimation, acting in all of these cases as a transcriptional activator (Pflock et al., 2005; Pflock et al., 2006; Wen et al., 2007; Loh et al., 2010). Deletion of ArsS impairs colonization of the murine stomach, but this HK is not essential for cell viability under neutral pH (Panthel et al., 2003; Loh and Cover, 2006). In contrast, ArsR appears essential for *in vitro* growth of *H. pylori*, suggesting that the expression of certain essential genes depends on the non-phosphorylated form of ArsR (Beier and Frank, 2000; Gupta et al., 2009). ArsRS is also involved in the acid-dependent negative regulation of the expression of adhesins and biofilm formation (Goodwin et al., 2008; Servetas et al., 2016; Acio-Pizzarello et al., 2017; Servetas et al., 2018), and contributes to acetone metabolism, resistance to oxidative stress and quorum-sensing (Loh et al., 2010).

*H. pylori* expresses two other TCRS involving two-partner proteins that play relevant roles for the *in vitro* growth and pathogenicity of the microorganism. Thus, the CrdRS system, composed of the CrdS HK and the OmpR-like CrdR RR appears to be involved in copper homeostasis (Waidner et al., 2005) and survival of the pathogen under nitrosative stress (Hung et al., 2015). On the other hand, the regulatory partner composed of the cytoplasmic sensor FlgS HK and the NtrC-like FlgR RR mediates the expression of the σ^54^-dependent flagellar genes that encode structural proteins of the basal body and hook, which are essential for *H. pylori* motility (Spohn and Scarlato, 1999; McDaniel et al., 2001; Brahmachary et al., 2004). The internal signal sensed by FlgS that triggers its autophosphorylation and subsequent activation of FlgR remains unknown (Alvarado et al., 2019).

Along with the aforementioned typical TCRS, the regulatory machinery of *H. pylori* includes two orphan OmpR-like RRs, the proteins HP1043 (also known as HsrA) and HP1021 (Beier and Frank, 2000). HsrA is a homodimeric essential RR that lacks the conserved phosphorylation site and appears to act as a phosphorylation-independent transcriptional activator (Schar et al., 2005; Hong et al., 2007; Pelliciari et al., 2017; Zannoni et al., 2021). The protein regulates its own expression (Delany et al., 2002), but also the expression of a plethora of other genes involved in crucial cellular processes such as translation, transcription, energy metabolism, nitrogen metabolism, chemotaxis, and redox homeostasis (Olekhnovich et al., 2013; Olekhnovich et al., 2014; Pelliciari et al., 2017).

The HP1021 orphan RR acts as an inhibitor of DNA replication by binding to specific sequences within the *oriC* region, thereby avoiding DnaA-*oriC* interactions and the unwinding of duplex DNA (Donczew et al., 2015). The DNA binding activity of this RR is affected by both the redox state of its cysteine residues and the presence of divalent metal cations, especially Zn^2+^ (Szczepanowski et al., 2021). Hence, the regulator functions as a redox sensor that controls DNA replication, synthesis of Fe-S clusters, acetone metabolism, and response to oxidative stress (Pflock et al., 2007; Donczew et al., 2015; Szczepanowski et al., 2021). Deletion mutants of this dispensable protein exhibit a small-colony phenotype (McDaniel et al., 2001).

*H. pylori* exhibits chemotactic responses to a variety of chemical cues, which are perceived by a battery of chemoreceptors (Machuca et al., 2017; Johnson and Ottemann, 2018). The transduction of the signal from the receptors to the flagellar motor is mediated by two CheY response regulators, the protein CheY1 and the bifunctional protein CheY2/A, which consists of a CheY-like receiver domain (CheY2) fused to the CheA HK (Foynes et al., 2000; Jimenez-Pearson et al., 2005). Mutants in any one of these two proteins exhibited impaired chemotaxis and failed to colonize animal models (Foynes et al., 2000).

### Campylobacter jejuni

*C. jejuni* is able to spatially discriminate among different regions of the host intestine by monitoring short-chain fatty acids (*e.g*. butyrate, acetate) and the lactate produced by the commensal microbiota of lower and upper intestinal tracks, respectively (Luethy et al., 2017; Goodman et al., 2020). The BumSR TCRS perceives butyrate levels and triggers a dual effect on gene expression, activating the transcription of a set of genes but repressing the transcription of other targets (Luethy et al., 2015; Goodman et al., 2020). BumR appears relevant for colonization of both avian hosts and humans (Crofts et al., 2018; Goodman et al., 2020). BumS lacks transmembrane domains and *in vitro* autokinase activity, but this cytoplasmic protein exhibited a robust and specific phosphatase activity for BumR (Goodman et al., 2020). Given that phosphorylation appears to increase the *in vitro* DNA-binding affinity of BumR by its target promoters, BumS could function as a negative modulator of its cognate RR (Goodman et al., 2020). BumSR modulates the expression of genes involved in basic metabolism, iron/heme acquisition, respiration, and some colonization factors (Luethy et al., 2015).

The RacSR TCRS modulates gene expression in response to changes in temperature, acting as both an activator and a repressor of transcription (Bras et al., 1999). Inactivation of both RacS and RacR significantly reduced chicken colonization and led to defective growth above 42°C; however, both mutants displayed only minimal defects in their *in vitro* growth at 42°C (Bras et al., 1999; Apel et al., 2012). Impairment in chicken colonization appeared to be a consequence of multiple phenotypic changes, including misregulation of several heat shock proteins but also alterations in cellular morphology that diminished motility and reduced epithelial cell invasion (Apel et al., 2012). RacSR has also been associated with glutamine catabolism (van der Stel et al., 2015b), and the induction of changes in the respiratory metabolism (van der Stel et al., 2015a).

CprRS is a TCRS composed of the essential OmpR-like response regulator CprR and the dispensable CprS HK. Mutants of CprS appeared defective in chick colonization with an increased susceptibility to osmotic and oxidative stress (Svensson et al., 2009). Little is known about the CprRS regulon (Svensson et al., 2009; Svensson et al., 2015), but the *in vitro* essentiality of the RR and the non-essential nature of its cognate HK resemble the regulatory scenario observed in the *H. pylori* TCRS ArsRS. However, a point mutation in the phosphoacceptor Asp52 residue of the CprR REC domain impaired viability, indicating that CprR phosphorylation is essential despite the dispensability of CprS (Svensson et al., 2015).

In DccRS, the OmpR-like DccR RR is specifically phosphorylated by its cognate DccS HK in the late stationary growth phase, probably using released metabolic products as triggering signals (Wosten et al., 2010). The activated RR enhances transcription of a handful of genes encoding putative periplasmic and membrane proteins that appeared relevant, but not essential for chicken colonization (Wosten et al., 2010). Deletion mutants of DccR and DccS exhibited reduced colonization in mice and chicks, but this TCRS is dispensable for *in vitro* growth (MacKichan et al., 2004).

The *C. jejuni* TCRS comprising the partners PhosS and PhosR enables survival of this food-borne pathogen outside their hosts, in phosphate-deprived environments such as surface waters (Wosten et al., 2006). Limitation of inorganic phosphate is perceived by the PhosS HK, which autophosphorylates itself and transfers the phosphoryl group to the receiver domain of PhosR. The activated RR enhances the expression of a phosphate-inducible regulon, mostly composed by genes encoding phosphate uptake mechanisms. Both the *in vitro* growth under phosphate-replete conditions and the chicken colonization capability were similar in a *phosR* mutant and its isogenic wild-type strain. (Wosten et al., 2006).

Similarly to *H. pylori*, transcription of genes encoding structural proteins of the basal body and hook of *C. jejuni* flagellum, but also the minor filament protein FlaB, is regulated by σ^54^ factor (RpoN), whose function is stimulated by the FlgSR TCRS (Wosten et al., 2004). A signal associated to the flagellar type III secretion system (T3SS) in conjunction with the flagellar proteins FliG (C ring) and FliF (MS ring) is presumed to trigger autophosphorylation of the cytoplasmic FlgS HK, which subsequently activates its cognate FlgR RR (Joslin and Hendrixson, 2009; Boll and Hendrixson, 2013). Curiously, the *C. jejuni* FlgR RR undergoes an unusual phase-variable production that affects σ^54^-dependent expression of flagellar genes and consequently the motility and colonization capabilities of the bacterial cell. By this mechanism, a small amount of colonizing *C. jejuni* cells become non-motile and they would be more readily shed into the environment, thereby promoting transmission and colonization of new naïve hosts (Hendrixson, 2006).

Besides the above-described typical TCRS, *C. jejuni* expresses two relevant Omp-like orphan RRs. Among them, it is worth noticing CosR, the essential orthologue of the *H. pylori* HsrA RR (Raphael et al., 2005; Muller et al., 2007). CosR appears to modulate the expression of several genes involved in a broad variety of cellular processes, including oxidative stress defenses, respiration, energy metabolism and biosynthesis, gene regulation, copper tolerance, flagellum biosynthesis, lipid metabolism, biofilm formation, among others (Hwang et al., 2011; Hwang et al., 2012; Turonova et al., 2015; Park et al., 2021). This RR exhibits a dual role on gene expression, repressing the transcription of several genes involved in the oxidative stress response such as SodB, Dps, Rrc and LuxS, but acting as an activator of AhpC (Hwang et al., 2011) and KatA (Hwang et al., 2012). Additionally, CosR represses the transcription of CmeABC (Hwang et al., 2012; Grinnage-Pulley et al., 2016), the major multidrug efflux pump of *C. jejuni* (Sharifi et al., 2021).

A second *C. jejuni* orphan RR, the protein CbrR, is an atypical RR that lacks a DNA-binding domain, but contains a C-terminal GGDEF motif commonly observed in diguanylate cyclase enzymes (Raphael et al., 2005; Cox et al., 2022). Deletion mutants of CbrR exhibited increased sensitivity to sodium deoxycholate, enhanced motility and reduced ability to colonize chickens (Raphael et al., 2005; Cox et al., 2022), whereas CbrR overexpression resulted in loss of flagella and a significant reduction in biofilm formation (Cox et al., 2022). Yeast two-hybrid screenings have showed that CbrR interacts with CheA (Parrish et al., 2007), which seems to indicate a putative role in chemotaxis.

## TCRS as targets for novel antimicrobials against *H. pylori and C. jejuni*

Bacterial TCRS have been proposed as promising therapeutic targets due to their validated essentiality for growth and pathogenicity of most clinical relevant pathogens (Bem et al., 2015; González et al., 2018; Hirakawa et al., 2020; Roncarati et al., 2022). TCRS become attractive targets for drug development against *H. pylori* and *C. jejuni*, where these regulatory proteins modulate essential physiological processes such as transcription, translation, energy metabolism and redox homeostasis, but also relevant mechanisms for host colonization and persistence of *in vivo* infection including cell morphology, motility, chemotaxis, acid acclimation as well as the expression of several virulence factors.

At least two RRs appear essential for *H. pylori* viability, ArsR from the pH-sensitive ArsRS TCRS, and the orphan OmpR-like effector protein HsrA. Similarly, two RRs have proved to be indispensable for *C. jejuni* survival, CprR from the CprSR TCRS, and the orphan regulator CosR. In addition, a group of other HKs and RRs are essential for optimal host colonization such as ArsS in *H. pylori*, which is indispensable for acid acclimation and gastric colonization, or the FlgSR TCRS in both pathogens, whose deletion results in loss of motility and chemotaxis. However, even though the expression of a variety of essential TCRS-related proteins in *H. pylori* and *C. jejuni* opens the door for the promising use of novel effective therapeutic targets in drug development; studies focused on this topic are relatively scarce.

To date, some investigations have been carried out in order to validate HsrA and ArsR as effective therapeutic targets against *H. pylori* infection as well as discovering low-molecular weight inhibitors of these RRs exhibiting strong bactericidal activities against antibiotic-resistant strains of this pathogen (González et al., 2019a; González et al., 2019b; González et al., 2020; Antoniciello et al., 2022; González et al., 2022). Notably, some of the novel antibacterial candidates evaluated in these studies, including natural flavonoids and 1,4-dihydropiridines, have shown *in vitro* antimicrobial activities in the range of those observed with first-line conventional antibiotics (González et al., 2021; González et al., 2022). In some cases, these novel antimicrobial candidates exhibited synergistic effects in combination with conventional antibiotics, leading to reversion of drug-resistant phenotypes (González et al., 2019b), but also showed minor antimicrobial actions on representative members of human normal microbiota (González et al., 2022).

## Future perspectives

The rapid emergence and dissemination of multidrug-resistant strains worldwide has effectively diminished the efficacy of current antibiotic therapies against most clinically relevant pathogens, including *H. pylori* and *C. jejuni*. A major challenge in the R&D of new effective antibiotics is the identification of novel molecular targets that allow for overcoming the current circulating resistome. The use of essential TCRS as targets for novel antimicrobials and antivirulence strategies against major pathogenic species of *Campylobacterota* could be a gateway to novel personalized combinatory therapies that can make it possible to cope with the increasing concern of multidrug resistance. Targeting TCRS-related proteins uniquely found in this group of microorganisms would enable the development of next-generation precision antibiotics, with highly selective antimicrobial action and minor risk of dysbiosis.

## Author Contributions

JC, AL, and AG wrote the manuscript. All authors contributed to the article and approved the submitted version.

## Funding

This work has been supported by the Government of Aragon, Spain (B25_20R) and University of Zaragoza (2018/0420).

## Conflict of interest

The authors declare that the research was conducted in the absence of any commercial or financial relationships that could be construed as a potential conflict of interest.

## Publisher’s note

All claims expressed in this article are solely those of the authors and do not necessarily represent those of their affiliated organizations, or those of the publisher, the editors and the reviewers. Any product that may be evaluated in this article, or claim that may be made by its manufacturer, is not guaranteed or endorsed by the publisher.
